# Middle East respiratory syndrome coronavirus (MERS-CoV) neutralising antibodies in a high-risk human population, Morocco, November 2017 to January 2018

**DOI:** 10.2807/1560-7917.ES.2019.24.48.1900244

**Published:** 2019-11-28

**Authors:** Anass Abbad, Ranawaka APM Perera, Latifa Anga, Abdellah Faouzi, Nhu Nguyen Tran Minh, Sk Md Mamunur Rahman Malik, Nadia Iounes, Abderrahmane Maaroufi, Maria D Van Kerkhove, Malik Peiris, Jalal Nourlil

**Affiliations:** 1 Medical Virology and BSL-3 Laboratory, Institut Pasteur du Maroc, Casablanca, Morocco; 2 Laboratoire d’Ecologie et d’Environnement, Faculté des Sciences Ben M’Sik, Université Hassan II, Casablanca, Morocco; 3 School of Public Health, University of Hong-Kong, Hong Kong SAR, China; 4 Health Emergencies Programme, World Health Organization, Regional Office for the Eastern Mediterranean, Cairo, Egypt; 5 Health Emergencies Programme, World Health Organization, Geneva, Switzerland; 6 HKU-Pasteur Research Pole, University of Hong Kong, Hong Kong SAR, China; 7 These authors contributed equally to this work

**Keywords:** Middle East Respiratory Syndrome Coronavirus, MERS-CoV, dromedaries, Morocco, zoonosis, transmission

## Abstract

**Background:**

Middle East respiratory syndrome coronavirus (MERS-CoV) remains a major concern for global public health. Dromedaries are the source of human zoonotic infection. MERS-CoV is enzootic among dromedaries on the Arabian Peninsula, the Middle East and in Africa. Over 70% of infected dromedaries are found in Africa. However, all known zoonotic cases of MERS have occurred in the Arabian Peninsula with none being reported in Africa.

**Aim:**

We aimed to investigate serological evidence of MERS-CoV infection in humans living in camel-herding areas in Morocco to provide insights on whether zoonotic transmission is taking place.

**Methods:**

We carried out a cross sectional seroprevalence study from November 2017 through January 2018. We adapted a generic World Health Organization MERS-CoV questionnaire and protocol to assess demographic and risk factors of infection among a presumed high-risk population. ELISA, MERS-CoV spike pseudoparticle neutralisation tests (ppNT) and plaque neutralisation tests (PRNT) were used to assess MERS-CoV seropositivity.

**Results:**

Serum samples were collected from camel slaughterhouse workers (n = 137), camel herders (n = 156) and individuals of the general population without occupational contact with camels but living in camel herding areas (n = 186). MERS-CoV neutralising antibodies with ≥ 90% reduction of plaque numbers were detected in two (1.5%) slaughterhouse workers, none of the camel herders and one individual from the general population (0.5%).

**Conclusions:**

This study provides evidence of zoonotic transmission of MERS-CoV in Morocco in people who have direct or indirect exposure to dromedary camels.

## Introduction

The Middle East respiratory syndrome coronavirus (MERS-CoV) is an emerging virus of great global public health concern [1,2]. From its initial recognition in 2012 in Saudi Arabia [3] to August 2019, there have been 2,468 patients with confirmed MERS-CoV infection, including 850 deaths, reported to the World Health Organization (WHO) from 27 countries [4]. Dromedary camels are known to be the source of human infection [5]. The virus is enzootic in dromedaries in the Arabian Peninsula, the Middle East, many regions of Africa, as well as Pakistan and Bangladesh. Over 70% of the global population of infected dromedaries are found in Africa, including Morocco [6,7]. Although travel-associated cases have been reported from several countries, zoonotic MERS cases have only been reported in the Arabian Peninsula and the Middle East [2]. The reasons for the apparent absence of zoonotic MERS in Africa in spite of exposure to virus-infected dromedaries is unclear, but likely because of several factors [2]. This may be owed to genetic and phenotypic differences in virus strains circulating in Africa [8], behavioural factors relating patterns of exposure, or alternatively, that MERS is not being detected because of the assumption that zoonotic MERS does not occur in Africa which may lead to a lack of testing for MERS-CoV.

Humans with regular contact with dromedaries in the Arabian Peninsula have higher seroprevalence to MERS-CoV than the general population [9]. Thus far, only a limited number of serological studies have been conducted in people with intense exposure to MERS-CoV-infected camels in Africa. Such studies are, however, important to better understand the geographic extent of MERS-CoV infection in human populations. Camel-exposed abattoir workers in Nigeria had no serological evidence of MERS-CoV infection despite intensive exposure to MERS-CoV infected camels [10]. Similarly, people in contact with camels in Kenya and Egypt had no serological evidence of MERS-CoV infection [11-13]. Another Kenyan study of 1,122 individuals without direct occupational exposure to dromedary camels found two individuals with low levels of neutralising antibody to MERS-CoV; to our knowledge, these are the only known instances of MERS-CoV seropositivity in humans in Africa prior to this study [14].

In Morocco, a recent study of MERS-CoV showed seroprevalence ranging from 48.3% to 100% and viral RNA shedding rates ranging from 0% to 7.6% among dromedaries [6]. Southern Morocco is the region of the country with the highest density of dromedary camels [15]. People living in this region share a close cultural and economic bond with dromedaries, and they are also consumers of dromedary milk, meat and other products. There are with many people having close and repetitive contact with dromedaries, including slaughterhouse workers, camel market workers and camel herders.

Since there is no information on MERS-CoV infections in humans in Morocco, the aim of this study was to determine the MERS-CoV seroprevalence among a presumed high-risk population, people in close contact with dromedaries, that is to say, those living in camel herding areas, including individuals with occupational exposure to dromedaries.

## Methods

We adapted and used an existing investigation protocol for cross-sectional seroepidemiologic study of MERS-CoV infection in high-risk populations in contact with dromedary camels developed by WHO [16]. The WHO protocol was translated into French, questions were modified, deleted or added to make it more relevant to Morocco, and it was used to ascertain risks of exposure to dromedaries as well as dromedary products.

### Setting and study population

The administrative regions of Guelmim-Oued Noun, Laâyoune-Sakia El Hamra and Dakhla-Oued Ed-Dahab were selected for study because 83.2% of all dromedary camels in Morocco are in these three regions [15]. The population of these regions was considered at high-risk of MERS-CoV infection because of direct or indirect contact with dromedaries, and for the study purposes, further divided into in three categories; camel herders and slaughterhouse workers, both with occupational exposure, and the general population, i.e. individuals living in high-risk areas who did not have direct occupational contact with dromedaries.

### Field sampling

Human blood sample collection was carried out over the 3 months from November 2017 through January 2018. A convenience sampling strategy was used to sample the different risk groups which led to blood samples being collected from 156 camel herders, 137 camel slaughterhouse (abattoir) workers and 186 individuals from the general population. Each participant provided signed informed consent and were administered a detailed questionnaire to elicit demographic information, as well as information about food habits, duration and type of exposure to camels and history of respiratory symptoms. For camel herders and abattoir workers, the use of personal protective equipment such as boots, coveralls, masks, gloves or coats, or other hygiene practices was also elicited.

Two hundred age-stratified sera collected in 2017 from blood donors in Hong Kong aged 16 to 69 years with no known exposure to dromedary camels were used as controls.

### Virological analysis

Human sera were tested for MERS-CoV IgG antibodies using a MERS-CoV S1 spike ELISA (EI 2604–9601 G kit, Euroimmun, Lübeck, Germany) according to the manufacturer’s instructions, at the Medical Virology and BSL-3 Laboratory at Institut Pasteur du Maroc, Casablanca, Morocco. The extinction value of the calibrator included in the test defines the upper limit of the reference range in non-infected humans and this value was set as the cut-off. The ELISA was made semi-quantitative by calculating the ratio of the extinction value of the serum sample over the extinction value of the calibrator. The manufacturer recommends cut-off ratios of < 0.8 be interpreted as negative, ≥ 0.8 and < 1.1 as borderline, and ≥ 1.1 as positive. Because subsequent publications suggested a lower ELISA cut-off of ratio ≥ 0.3 for screening purposes for the selection of sera to be confirmed by neutralisation tests, we have also included ELISA optical density (OD) ratios of ≥ 0.3 in our analysis [9].

All sera were also screened in triplicate in a MERS-CoV pseudoparticle neutralisation test (ppNT) as described previously [11]. All sera positive at a ppNT screening dilution of 1:10 were titrated to end-point in the ppNT assay, as well as in a plaque reduction neutralisation test (PRNT) conducted in BSL-3 containment. The end-point for the ppNT assay was the highest serum dilution giving a ≥ 90% reduction in the luciferase signal compared with negative control. The end-point in the PRNT was the highest serum dilution that gave ≥ 50% (PRNT_50_) or ≥ 90% (PRNT_90_) reduction of virus plaques compared with control. The methods have been described elsewhere [11,17].

Sera-positive at a titre of ≥ 1:20 in ppNT and ≥ 1:10 in PRNT_90_ assays were regarded as positive. Sera-positive at a titre of ≥ 1:20 in ppNT and ≥ 1:10 in PRNT_50_, but negative in PRNT_90_ assays were regarded as a borderline positive neutralisation result. All other sera were regarded as negative.

### Statistical analysis

Statistical significance of serological positivity by exposure group was analysed using the chi-squared test with Yates correction. Statistical significance was defined as p < 0.05.

### Ethical approval

This study was approved by the national Ethical Committee of Biomedical Research (CERB), Université Mohammed V, Faculté de Médecine et de Pharmacie de Rabat (reference number 55/16). Age-stratified sera from Hong Kong blood donors previously obtained as part of other studies were used as controls. The anonymised use of these sera for sero-epidemiological studies was approved by The University of Hong Kong and the Hospital Authority (Hong Kong West) institutional review board (UW 16-254).

## Results

### Demographics of the study group

Samples were collected from a total of 479 people, of whom 120 were female and 359 were male (Table 1). Of study participants, 143 were ≤ 30 years of age (range: 16–30), 140 came from the Guelmim-Oued Noun region, 200 from the Laâyoune-Sakia El Hamra region and 139 from the Dakhla-Oued Ed-Dahab region. The male-to-female ratio of the camel herders was 11:1, for slaughterhouse workers it was 26:1 and for the general population it was 0.8:1 (Table 1).

**Table 1:** Middle East respiratory syndrome coronavirus (MERS-CoV) study population demographics and dromedary exposure by study group, Morocco, 2017–2018 (n = 479)

Characteristics	Camel herders (n = 156)		Slaughterhouse workers (n = 137)		General population (n = 186)		Total
	n	%	n	%	n	%	
**Sex**							
Male	143	92	132	96	84	45	359
Female	13	8	5	4	102	55	120
**Age**							
≤ 20 years	8	5	16	12	11	6	35
21–30 years	29	19	33	24	46	26	108
31–40 years	20	13	26	19	38	20	84
41–50 years	34	22	35	26	32	17	101
51–60 years	39	25	20	15	31	17	90
61–70 years	24	15	3	2	22	12	49
> 70 years	2	1	4	3	6	3	12
**Dromedary camels kept around home**							
Presence of camels	95	61	28	20	10	5	133
**Contact with camels kept around home**							
Direct, recurrent contact	95	61	26	19	6	3	127
Indirect contact	61	39	111	81	180	96	352
**Consumption of raw camel meat**							
> once per week	86	55	59	43	34	18	179
< once per week–once per month	22	14	5	4	3	2	30
< once per month–once in past 6 months	18	12	8	7	22	12	48
None	30	19	63	46	127	68	220
**Consumption of camel urine**							
> once per week	23	15	13	9	0	0	36
< once per week–once per month	10	6	2	1	2	1	14
< once per month–once in past 6 months	53	34	6	4	10	5	69
None	70	45	116	85	173	93	359
**Consumption of unpasteurised camel milk**							
> once per week	73	47	53	39	41	22	167
< once per week–once per month	7	4	49	36	27	15	83
< once per month–once in past 6 months	42	27	7	5	50	27	99
None	34	22	28	20	68	37	130

### Exposure to dromedaries and dromedary products

The direct and indirect exposure patterns to dromedaries and dromedary products (raw camel meat, camel urine or unpasteurised camel milk) of the study subjects are summarised in Table 1. Consumption of raw camel meat and unpasteurized camel milk was not uncommon in all three groups, ranging between 32% and 81%, and 63% and 80%, respectively (Table 1). None of the participants had a history of severe pneumonia that required hospital admission and none of them had travelled outside of Morocco.

### Camel herders

Camel herders (n = 156) had been such for at least 1 year, travelling with nomadic dromedary herds for many months of the year with 95 (61%) and having contact with camels around their home the rest of the year. All herders had camels in their herds, but 5 (3%) also had sheep and goats within their herd. Their tasks while herding included cleaning and caring for the animals (n = 113; 72%), feeding, milking, marketing and other supplemental tasks (n = 43; 28%). Most herders reported contact with dromedary waste (n = 140; 90%), while some had contact with ill (n = 52; 33%) or dead (n = 21; 13%) dromedaries, and some directly handled dromedaries that died from illness (n = 19; 12%). Personal protective gear, e.g. boots, coverall, mask or coat, were used only by 4 (3%) of the herders.

### Slaughterhouse workers

The slaughterhouse workers (n = 137) had worked in the dromedary slaughterhouse for at least 1 year. Their working hours were 5 to 7 hours per day, 6 days per week. They uniformly had occupational contact with camels at the slaughterhouse, but some (n = 28; 20%) also had dromedaries around the home (Table 1). For 115 (84%), work included slaughtering, cleaning the slaughterhouse and storing the camel meat. The other 22 (16%) were not directly involved in slaughtering but involved in cleaning, transporting products and security. Personal protective gear, usually reported as boots, but not gloves or masks, was used by 117 (85%) of the slaughterhouse workers. Twenty-two (16%) regularly lived or slept in the slaughterhouse at least once a week.

### General population

Of the general population group (n = 186), 5% had dromedaries around the home, 3% had direct, recurrent contact with dromedaries, 18% frequently consumed raw camel meat and 22% consumed unpasteurised camel milk more than once per week .

### Virological findings

We tested 479 human sera for anti-MERS-CoV S1 IgG antibodies by ELISA. Using the ELISA kit recommended cut-offs, 20 sera (4.2%) were reactive with OD ratio of ≥ 1.1 and 21 (4.4%) were borderline reactive with OD ratio of ≥ 0.8 to < 1.1 (Table 2). Using the lower screening cut-off recommended by Muller et al. [9], 173 additional sera would be regarded as suspected positives requiring testing by a neutralisation test. We tested all 479 sera irrespective of ELISA results for MERS-CoV neutralising activity using the ppNT assay; three (0.6%) were positive at a ppNT titre of ≥ 1:20 while one (0.2%) was positive at a ppNT titre of 1:10 (Table 3). Two of these were positive by ELISA (OD ratio cut-off ≥ 1.1), one borderline (OD ratio 0.83) and the other negative (OD ratio of 0.47) as per kit instructions, but would be recommended for confirmatory testing in the algorithm used by Muller et al. [9]. Of these four ppNT-positive sera at a dilution of 1:10, three (0.6%) were confirmed with PRNT_90_ reactivity, regarded as confirmed neutralising sera, while the one with a ppNT 1:10 and ELISA OD ratio of 0.83 reduced plaque numbers by between 50% to 90%, i.e. positive in PRNT_50_ but not PRNT_90,_ and regarded as borderline neutralising in the confirmatory test (Table 2).

**Table 2:** Middle East respiratory syndrome coronavirus (MERS-CoV) seroprevalence in groups by dromedary exposure, Morocco, 2017–2018 (n = 479)

Result	Camel herders (n = 156)		Slaughterhouse workers (n = 137)		General population (n = 186)		Statistical significance for association between seropositivity and exposure (chi-squared test)
	n	%	n	%	n	%	
**MERS-CoV S1 ELISA^a^**							
Positive	3	1.9	9	6.6	8	4.3	Chi-squared 6.1 p = 0.195 Not significant
Borderline	9	5.8	7	5.1	5	2.7	
Negative	144	92.3	121	88.3	173	93.0	
**Neutralisation tests^b^**							
Positive	0	0	2	1.5	1	0.5	Chi-squared 0.92 p = 0.63 Not significant
Borderline	0	0	1	0.7	0	0	
Negative	156	100	134	97.8	185	99.5	

^a^ ELISA positive, borderline and negative was defined as recommended in the test kit.

^b^ Positives defined as ppNT-positive and confirmed by ≥ 90% reduction of plaque counts in PRNT test; borderline is defined as ppNT-positive at serum dilution of 1:10 but only reduced plaque counts between 50 and 89% in PRNT tests; negatives defined as negative for neutralising antibody in the screening ppNT assay at a dilution of 1:10.

**Table 3:** Characteristics, exposure and other risk factors of people seropositive for Middle East respiratory syndrome coronavirus (MERS-CoV) by neutralisation tests, Morocco, 2017–2018 (n = 4)

Sample location	Age group	Sex	MERS-CoV antibody results				Dromedary exposure	Other relevant risk factors
			ELISA OD ratio (and results)	ppNT titre	PRNT_50_ titre	PRNT_90_ titre		
Laâyoune-Sakia El Hamra Region	41–50 years	Male	0.83 (borderline)^a^	1:10	1:10	< 1:10	Slaughterhouse worker, 7 years. Slaughtering and cleaning. Wears boots, coveralls, gloves at work.	Others in household have regular contact with camels. Consume camel urine at least once per week,
Laâyoune-Sakia El Hamra Region	> 70 years	Male	1.1 (positive)^a^	1:40	1:20	1:10	Slaughterhouse worker, 25 years. Slaughterer. No personal protective equipment at work.	Drinks camel urine at least once per month.
Guelmim-Oued Noun Region	31–40 years	Male	1.59 (positive)^a^	1:40	1:20	1:10	Slaughterhouse worker, 17 years. Slaughtering and cleaning, product storage. Boots while at work.	Others in household have regular camel exposure. Regularly (at least once per week) consumes camel urine, unpasteurised milk, consumes raw camel meat. Smoker.
Guelmim-Oued Noun Region	51–60 years	Female	0.47 (negative)^a^	1:40	1:20	1:10	General population. No direct camel exposure.	Others in household have regular camel exposure. Regularly (at least once per week) consumes camel urine and raw unpasteurised milk. Has diabetes and hypertension.

OD: optical density.

^a^ All ELISA results are defined on the basis of the manufacturer-recommended OD ratio cut-off values. Notably, if the OD ratio cut-off suggested by Muller et al. [9], which is  ≥ 0.3, is used, all four sera would be picked for confirmatory testing in neutralisation tests if ELISA was used as the sole screening test. In this study, all sera were tested by ppNT neutralization tests irrespective of ELISA result.

The 200 sera from Hong Kong serving as negative controls were all negative in PRNT_50_ and PRNT_90_ assays. Validation of the ppNT test with 528 negative control sera has been previously reported [11].

Of the 41 sera that were positive or borderline by ELISA, i.e had an OD ratio ≥ 0.8, 38 were negative by ppNT. The other 438 sera were negative by both tests. There was a significant association between the results of the two tests (chi-squared with Yates correction: 14.9; p = 0.0001). However, the scatterplot between the ELISA and ppNT assays did not reveal a high level of correlation (correlation coefficient R-value: 0.13; 95% CI: 0.039–0.22) (Figure). If we consider that sera positive in the screening ppNT assay and confirmed by PRNT_90_ as true MERS-CoV positive sera, ELISA with the cut off recommended by the manufacturer had a sensitivity of 66.7% and a positive predictive value (PPV) of 10%. The serum with borderline PRNT activity was also borderline in ELISA reactivity. If the lower OD ratio of ≥ 0.3 is used as the cut-off for selecting sera for confirmatory testing as recommended by Mueller et al. [9], then the sensitivity of the ELISA for screening for sera subsequently confirmed as positive or borderline neutralising positive was 100% but the PPV was only 0.19%.

**Figure:**
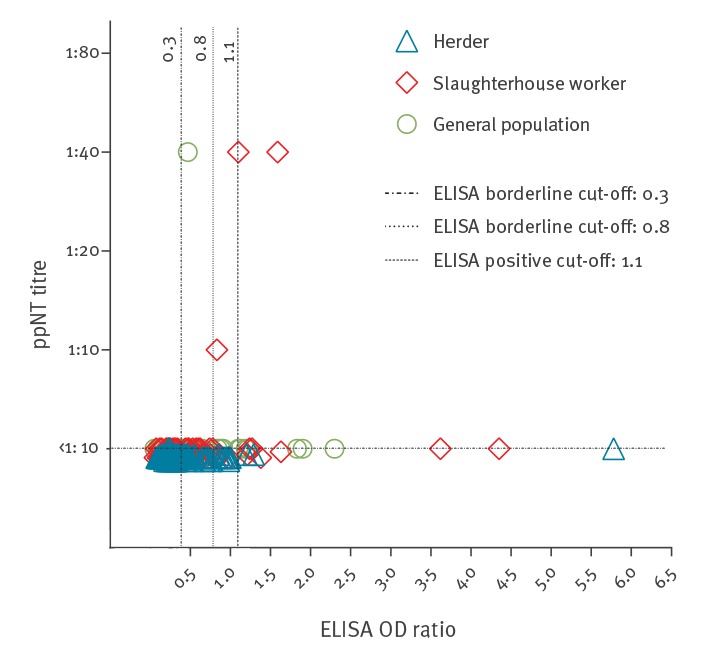
Scatter-plot showing correlation between ELISA OD ratios and the MERS spike pseudotype virus neutralisation antibody titres, Morocco, 2017–2018

MERS: Middle East respiratory syndrome; OD: optical density; ppNT: pseudoparticle neutralizisation tests.

OD ratios recommended as cut off for positive (1.1) and borderline (0.8) by the kit manufacturers are indicated. We also indicate OD ratio of 0.3, which has been recommended in some publications as a screening cut-off value [9]. Correlation coefficient R-value: 0.13 (95% confidence interval: 0.039–0.22).

Nine (6.6%) of 137 slaughterhouse workers, three (1.9%) of 156 camel herders and eight (4.3%) of 186 people from the general population were MERS-CoV antibody-positive in the ELISA test, using the ELISA kit recommended cut-off values (Table 2). Three (2.2%) of 137 slaughterhouse workers, none of 156 camel herders and one (0.5%) of 186 people from the general population were MERS-CoV neutralising antibody positive by ppNT assay. All four MERS-CoV neutralising antibody-positive sera also reduced virus plaque numbers by ≥ 50%; three of them reduced plaque counts by ≥ 90% (Table 2). There was no statistically significant association between exposure groups and MERS-CoV seropositivity by either ELISA or neutralisation tests. It should be noted all groups were resident in camel herding areas likely had some exposures to camels or camel products.

### Characteristics and risk factors of MERS-CoV-seropositive individuals

The three camel slaughterhouse workers who were confirmed (n = 2) or borderline (n = 1) neutralisation seropositive for MERS-CoV were males aged from 34 to 72 years. They had been working in the camel slaughterhouse for 7 to 25 years and regularly consumed camel urine. One regularly consumed unpasteurised camel milk and raw camel meat in addition (Table 3). The one person in the general population group who was seropositive for MERS-CoV was female in her mid-50s with diabetes and hypertension. She had no direct contact with camels, but others in the household had regular contact with camels. At least once a week, she consumed camel urine and raw unpasteurised camel milk (Table 3).

## Discussion

Our study provides the first serologic evidence of MERS-CoV infection in humans in Morocco, or anywhere in North or West Africa. The virus used for serology was the EMC strain of MERS-CoV because we have previously shown that there is no antigenic differences between MERS-CoV strain EMC and viruses from Africa [8]. The results suggest evidence of human infection, with two (1.5%) of the slaughterhouse workers having confirmed MERS-CoV PRNT_90_ neutralising antibody, one (0.7%) additional slaughterhouse worker with borderline PRNT_50_ neutralising activity and one (0.5%) person from the general population living in camel herding areas with confirmed PRNT_90_ neutralising antibody. The female from the general population group had confirmed serum MERS-CoV neutralising activity had no occupational or direct contact with camels but others in her household did, and she regularly consumed camel urine and unpasteurised camel milk. The role of camel products such as camel milk and urine in transmission of MERS-CoV has been previously raised, but remains unclear [18]. None of the seropositive people had travelled outside of Morocco suggesting that infection was acquired locally.

None of those with MERS-CoV antibodies had a history of severe pneumonia, a common feature of severe MERS [19]. This is not surprising as other serological studies in Saudi Arabia and Qatar have detected infection in people with no history of a MERS-like disease [9,20,21]. It is known that MERS-CoV infection may range from being asymptomatic to severe pneumonia, acute respiratory distress syndrome (ARDS) and death, and that some mild or asymptomatic infections may contribute to onward transmission [2,19,22].

Of the camel herders included in this study, none had confirmed serum MERS-CoV neutralising activity although we have previously found MERS-CoV circulating in camels from this region [6]. It is possible that herders are at lower risk than slaughterhouse workers because camels brought to slaughterhouses often transit via camel markets where mixing of camels from diverse sources allows for amplification of virus circulation which may result in higher rates of virus shedding and greater MERS-CoV exposure of these slaughterhouse workers.

In the three people with confirmed MERS-CoV neutralising antibody, the ppNT titres were 1:40 and PRNT_90_ titres were 1:10. This is not unexpected as even patients with confirmed MERS-CoV disease do not invariably mount robust neutralising or ELISA antibody titres unless they have severe pneumonia [23,24]. Thus, people with mild infections may have low or undetectable antibody titres to MERS-CoV, and furthermore, such antibody may wane over time. Serology may thus considerably under-estimate extent of mild or asymptomatic MERS-CoV infection, as has been previously suggested [20]. A recent study proposed that supplementing serology with MERS-CoV-specific CD4^+^ and CD8^+^ T-cell responses may identify additional individuals who have prior MERS-CoV infection in population based studies [20].

Previously published data on human MERS-CoV seroprevalence in the Arabian Peninsula [9,20,21,25-28], Africa [10-14] and Pakistan [29] are summarised (Supplementary Table S1). In the Arabian Peninsula, an aggregate of 1,090 people occupationally exposed to dromedaries have been tested with neutralisation tests for MERS-CoV antibody in seven studies and 68 (6.2%) were found to be seropositive. An aggregate of 1,200 human sera from people occupationally exposed to dromedaries have been previously tested in Africa (Egypt, Kenya and Nigeria) [10-14] with no seropositivity detected while 0.7% (2/293) people were seropositive in the present study. In the general population, MERS-CoV seroprevalence in the Arabian Peninsula was 0.15% of an aggregate of 10,365 persons sampled, 0.1% of 1,987 people tested in Kenya, Africa and 0.5% of 186 people in the present study in Morocco. See Supplementary Table S1 for more information.

While there was a significant association between the overall results of neutralisation and MERS S1 ELISA tests for detecting antibody to MERS-CoV in humans, the correlation between the two methods was poor. The sensitivity of ELISA using the kit recommended cut-off was 66.7% though it was 100% when the lower ELISA OD ratio suggested by Muller et al. [9] was used. However, the positive predictive value of MERS-CoV S1 antibody ELISA was poor with either cut-off when used for population seroepidemiology. Similar results have been reported in Saudi Arabia [20]. This needs to be considered when planning human seroprevalence studies.

## Conclusions

In conclusion, we found evidence of zoonotic transmission of MERS-CoV in Morocco, supporting previous data from Kenya [14] and indicating that MERS-CoV is capable of zoonotic infection in Africa. However, it is not yet known if these MERS-CoV circulating in Africa can cause zoonotic disease, i.e. invade the lower respiratory tract to cause severe viral pneumonia. The genetically diverse strains of MERS-CoV found in Moroccan dromedaries, as well as other regions of western Africa, e.g. Nigeria and Burkina Faso, might have a lower potential for severe zoonotic disease compared with the virus strain isolated in the Middle East [8]. Testing for MERS-CoV RNA in patients with severe acute respiratory infections in dromedary camel herding regions of Africa is needed to ascertain whether or not zoonotic MERS disease is occurring in Africa.

## Supplementary Data

535000 Supplementary Material
