# Supplementary material for: Middle East respiratory syndrome coronavirus (MERS-CoV) neutralising antibodies in a high-risk human population, Morocco, November 2017 to January 2018
Source: Euro Surveill. 2019 Nov 28;24(48):1900244. doi: 10.2807/1560-7917.ES.2019.24.48.1900244 (PMC6891945; doi:10.2807/1560-7917.ES.2019.24.48.1900244)
Supplement: Supplementary Material [file 1900244_PEIRIS_MERS_SupplementaryMaterial.pdf]

Supplementary Material

This supplementary material is hosted by *Eurosurveillance* as supporting information alongside the article 'Middle East respiratory syndrome coronavirus (MERS-CoV) neutralising antibodies in a high-risk human population, Morocco, November 2017 to January 2018', on behalf of the authors, who remain responsible for the accuracy and appropriateness of the content. The same standards for ethics, copyright, attributions and permissions as for the article apply. Supplements are not edited by *Eurosurveillance* and the journal is not responsible for the maintenance of any links or email addresses provided therein.

Supplementary table S1. Seroepidemiological studies in humans using neutralisation studies

Location (reference)	Camel exposed	Control
**Arabian Peninsula**		
Kingdom of Saudi Arabia [9].	2 (2.3%) of 87 camel herders<br>5 (3.6) of 140 camel abattoir workers	15 (0.15%) of 10,009 general population
Kingdom of Saudi Arabia [25].	0 of 300 camel workers	0 of 50
Kingdom of Saudi Arabia [26].	0 of 12 camel workers exposed to an infected herd<br>0 of 30 Vets in a camel hospital<br>0 of 3 camel abattoir workers	0 of 146
Kingdom of Saudi Arabia [28].	2 (0.9%) of 226 slaughterhouse workers	0 of 130 blood donors
Qatar [21]	2 (40%) of 5 camel abattoir workers<br>2 (9%)of 22 camel barn workers	
Kingdom of Saudi Arabia [20].	15 of 30 camel workers	0 of 30 controls
United Arab Emirates [27]	Three rounds of overlapping sampling was carried out. In the last round of sampling carried out in 2016–17, 40 of 235 camel workers sampled were seropositive.	
**Total**	**68 (6.2%) of 1090**	**15 (0.15%) of 10,365**
**Southern Asia**		
Pakistan [29]		0 of 2,409 general population in camel herding areas
**Africa**		
Egypt [12]	0 of 179 camel abattoir workers	
Egypt [11]		0 of 815 general population
Kenya [13]	0 of 760 camel exposed people	
Kenya [14]		2 (0.18%) of 1122 general population
Nigeria [10]	0 of 261 camel abattoir workers	0 of 50 abattoir workers not working with camels
**Total**	**0 of 1200**	**2 (0.1%) of 1987**
Morocco (this study)	2 (1.5%) of 137 camel abattoir workers<br>0 of 156 camel herders	1 (0.5%) of 186 general population
